# Identifying Mixed Mycobacterium tuberculosis Infection and Laboratory Cross-Contamination during Mycobacterial Sequencing Programs

**DOI:** 10.1128/JCM.00923-18

**Published:** 2018-10-25

**Authors:** David H. Wyllie, Esther Robinson, Tim Peto, Derrick W. Crook, Adebisi Ajileye, Priti Rathod, Rosemarie Allen, Lisa Jarrett, E. Grace Smith, A. Sarah Walker

**Affiliations:** a Nuffield Department of Medicine, John Radcliffe Hospital, Oxford, United Kingdom; b Public Health England Academic Collaborating Centre, John Radcliffe Hospital, Oxford, United Kingdom; c The National Institute for Health Research Health Protection Research Unit (NIHR HPRU) in Healthcare Associated Infections and Antimicrobial Resistance at University of Oxford, Oxford, United Kingdom; d Public Health England National Mycobacteriology Laboratory North and Central, Heartlands Hospital, Birmingham, United Kingdom; Virginia Commonwealth University Medical Center

**Keywords:** Mycobacterium tuberculosis, next-generation sequencing, quality control, mixed infection, cross-contamination

## Abstract

The detection of laboratory cross-contamination and mixed tuberculosis infections is an important goal of clinical mycobacteriology laboratories. The objective of this study was to develop a method to detect mixtures of different Mycobacterium tuberculosis lineages in laboratories performing mycobacterial next-generation sequencing (NGS). The setting was the Public Health England National Mycobacteriology Laboratory Birmingham, which performs Illumina sequencing on DNA extracted from positive mycobacterial growth indicator tubes. We analyzed 4,156 samples yielding M. tuberculosis from 663 MiSeq runs, which were obtained during development and production use of a diagnostic process using NGS. The counts of the most common (major) variant and all other variants (nonmajor variants) were determined from reads mapping to positions defining M. tuberculosis lineages. Expected variation was estimated during process development. For each sample, we determined the nonmajor variant proportions at 55 sets of lineage-defining positions. The nonmajor variant proportion in the two most mixed lineage-defining sets (F2 metric) was compared with that of the 47 least-mixed lineage-defining sets (F47 metric). The following three patterns were observed: (i) not mixed by either metric; (ii) high F47 metric, suggesting mixtures of multiple lineages; and (iii) samples compatible with mixtures of two lineages, detected by differential F2 metric elevations relative to F47. Pattern ii was observed in batches, with similar patterns in the M. tuberculosis H37Rv control present in each run, and is likely to reflect cross-contamination. During production, the proportions of samples in the patterns were 97%, 2.8%, and 0.001%, respectively. The F2 and F47 metrics described could be used for laboratory process control in laboratories sequencing M. tuberculosis genomes.

## Abstract (precis)

The detection of laboratory cross-contamination and mixed tuberculosis infections is an important goal of clinical mycobacteriology laboratories. The objective of this study was to develop a method to detect mixtures of different Mycobacterium tuberculosis lineages in laboratories performing mycobacterial next-generation sequencing (NGS).

## INTRODUCTION

Mycobacterium tuberculosis is an organism which has coevolved with humans during the early migrations of modern humans, diverging from a common M. tuberculosis ancestor about 75,000 years ago (1). Distinct lineages, corresponding to evolution occurring during these early migrations, are readily identified by next-generation sequencing (NGS), with each lineage characterized by ancient single-nucleotide variants (SNVs) which define deep branches in the M. tuberculosis phylogeny (1, 2).

### Multiple M. tuberculosis lineages.

Infection by multiple lineages of tuberculosis (TB) is well described and has been detected by observing mixed results on fractional sequencing (e.g., spoligotyping and mycobacterial interspersed repetitive-unit–variable-number tandem-repeat [MIRU-VNTR]) and validated by the characterization of multiple individual picks from solid medium (3). Multilineage infection is characterized by isolates differing by many hundreds of SNVs, in which respect it differs from the increasingly recognized and more common in-host microevolution (4). Reported rates of mixed infection vary markedly, as reviewed previously (3, 5), with rates between 10% and 30% reported in areas of current (5–7) or historical (8, 9) high prevalence. Much lower rates are reported in low-incidence countries (3), although systematic underdetection is likely to occur due to both the limited representation of bacteria in single-sputum samples of pulmonary disease and the decrease in diversity occurring during differential strain growth in broth culture (10).

### Implications of mixed infection.

Mixed infection, assessed by either MIRU-VNTR polymorphisms (7) or by heterogeneity in drug susceptibility testing (11), is independently associated with reduced drug treatment response, so there are compelling clinical reasons to try to identify it. There are also important technical implications of isolating mixed TB strains from a culture. First, such a finding may reflect cross-contamination within the laboratory (3). Second, mixed infection complicates the interpretation of drug resistance tests, whether phenotypic or genotypic, as one or other coinfecting strains may dominate the results from these tests. Third, it complicates the understanding of relatedness when techniques, such as SNV distance computation, are applied, as these generally assume that a single sequence is present when basecalling (5, 12–14), marking mixed sites as uncertain. Maximal likelihood tree drawing algorithms assume that such “uncertain” sites contain no information and impute a single nucleotide at each of such positions, an approach which may be inappropriate in the presence of mixtures.

Increasingly, NGS-based species and resistance determination is becoming routine in mycobacteriology laboratories and has been deployed in reference laboratories in England (15, 16). As part of the quality control and accreditation of the routine process now operating in these laboratories, we describe an approach to identifying mixed samples using Illumina next-generation sequencing data, illustrating its use by studying over 4,000 consecutive positive cultures from a single reference laboratory.

## MATERIALS AND METHODS

### Isolation of DNA from mycobacteria and sequencing.

**FIG 1:**
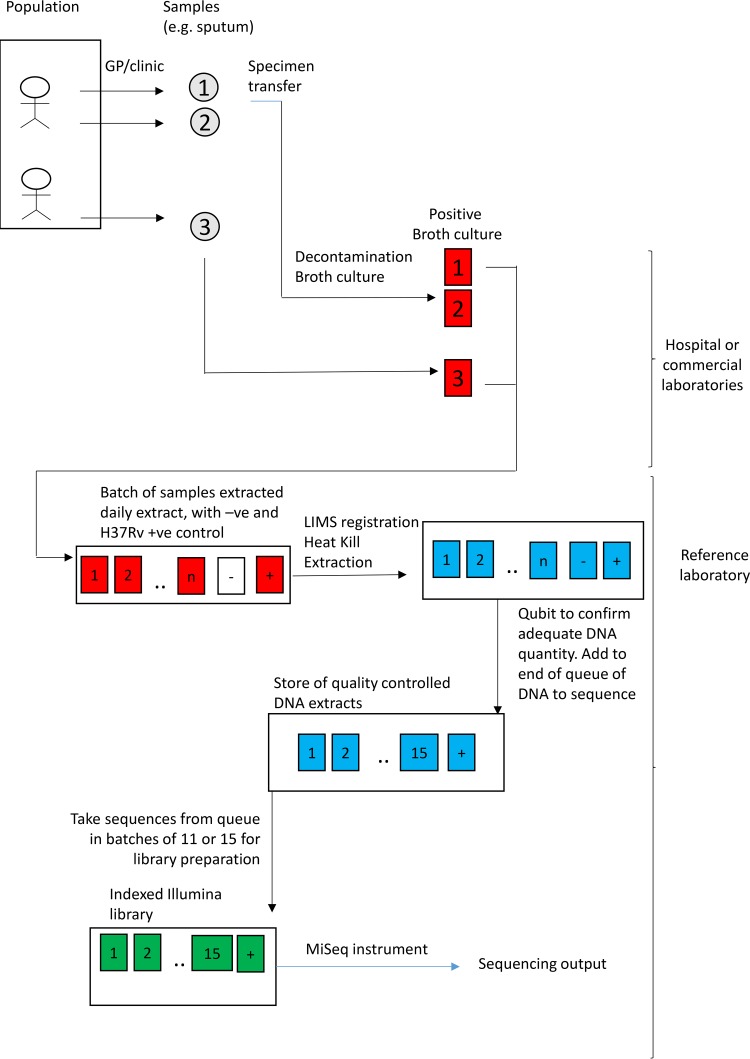
Laboratory and bioinformatic processing. GP, general practitioner; −ve, negative; +ve, positive; LIMS, lab information management system.

This study includes all mycobacteria processed between 01 June 2015 and 30 December 2017 in the Public Health England Midlands and North Reference Laboratory, whose catchment is approximately 15 million people, or about one-third of England. Clinical specimens were decontaminated and inoculated into mycobacterial growth indicator tubes (MGIT). During the process (Fig. 1), positive MGITs were batched when they became available, either following growth in the local laboratory or following receipt from another laboratory. Since a positive MGIT may contain more than one M. tuberculosis strain, and since subculture is not performed prior to DNA extraction and sequencing, the DNA extracted from such MGIT cultures may derive from multiple M. tuberculosis strains. Positive-control samples (H37Rv strain) were also grown in MGIT cultures. Batches of positive samples were extracted using a manual process, exactly as described in the supplemental methods in a previous study (16). Illumina sequencing libraries were prepared using Nextera XT chemistry from equal amounts of 12 (from 20 April 2015 to 31 July 2015) or 16 (from 01 August 2015 to 30 December 2017) mycobacterial DNA extracts (16), using manual steps (Table 1). Positive-control DNA (H37Rv, obtained from ATCC) was included as one of the 12 or 16 extracts in all libraries, either from a contemporaneously extracted broth culture or from stored DNA. Libraries were loaded into an Illumina MiSeq instrument and sequenced (16).

**TABLE 1:** Samples analyzed

Development stage	No. of sequences (samples and controls)	Date range (mo-day-yr)	No. of individuals providing samples	MiSeq run identifiers	No. of clinical samples	No. of MiSeq Runs
Development	938	04-20-2015 to 12-15-2015	630	101–291	776	154
Preproduction	1,167	04-01-2016 to 12-06-2016	753	1152–1522	919	163
Production	2,191	12-07-2016 to 12-30-2017	1,481	1523–2307	1,794	346

### Routine bioinformatic processing.

The routine bioinformatics pipeline deployed by Public Health England has been previously described (15). Briefly, reads were first processed using the Mykrobe predictor tool, which identifies Mycobacterium tuberculosis bacteria using species-specific k-mers (17). Specimens identified as containing M. tuberculosis bacteria were further processed (16) and mapped to the H37Rv v2 genome (NCBI RefSeq no. NC_000962.2) (18), as described previously (16), and vcf files were generated using SAMtools mPileup, with additional basecalling using GATK VariantAnnotator v2.1. A consensus base is called from high-quality bases provided one base accounts for >90% of the pileup; otherwise, the base is recorded as uncertain (“N”) (15).

### Estimation of the minor variant frequency within a set of lineage-defining positions.

In this study, we evaluated an additional step not present in the production pipeline. This evaluates high-quality base counts (identified by the BaseCounts VCF tag) at positions known to be lineage associated (2), which were extracted and summarized using code available at https://github.com/davidhwyllie/VCFMIX. We elected to study sites known to be subject to lineage-specific variation to maximize power in detecting interlineage mixtures, as nonlineage-associated sites can appear to be mixed for technical reasons, e.g., due to mismapping. Coll et al. (2) described the M. tuberculosis phylogeny and identified 62 sets of nucleotide positions defining the deep branches of the Mycobacterium tuberculosis lineage. At each position within a nucleotide set, in one particular clade, one nucleotide is uniquely present (i.e., is not present in any other of the known clades). These sets contain a median of 108 nucleotide positions (range, 1 to 898 positions). In this analysis, we considered 55 branches, excluding branches 1.2, 3.1, 3.1.2, 4.1.2, 4.3.4.2.1, 4.6, and 4.7 because they contain fewer than 20 positions, making estimates of minor variation in these positions less reliable than estimates in other branches.

The minor variant frequency at a set of bases can be due to sequencing error, mapping error, and/or bona fide interlineage mixtures (see Fig. S1A and B in the supplemental material). Minor variant frequencies were determined as follows: if there are *n* bases in a lineage-defining set, we count the high-quality depths (*d*) at each base, e.g., if *n* = 3 and *d*_1_ = 30, *d*_2_ = 70, and *d*_3_ = 100, the total depth (*D*) is

(1) $$D=\sum_{i=1}^{n}d_{i}=200$$

For each position, we also identify the most common base; the minor depth, *m*, is the total depth minus the most common base depth. If the minor depths are *m*_1_ = 3, *m*_2_ = 7, and *m*_3_ = 10, the total minor depth (*M*) is

(2) $$M=\overset{n}{\underset{i=1}{\sum }}m_{i}=20$$

We estimate the minor allele fraction *p* in the set as *M*/*D* = 0.1.

### F2 and F47 metrics.

If sequences from two different M. tuberculosis lineages are mixed together, the sets which uniquely define these lineages are mixed (Fig. S1C); there are a minimum of two and maximum of eight sets affected (e.g., a lineage 5/7 mixture mixes two sets of lineage-defining nucleotides, a 2.1/4.2.1 mixes five sets, and a 4.1.1.1/3.1.2.1 mixture mixes 8 sets). Only if more than two samples are mixed will more than 8 sets be mixed. In this work, we describe two metrics reflecting mixing. Having computed the minor allele frequency estimates of *p*_1_, *p*_2_, and *p*_55_, we can sort these in descending order, identifying the sets with the highest and lowest minor allele frequencies. We then estimate the minor variant frequency across the nucleotides in the top two (F2 metric) and lowest 47 (F47 metric) sets. For example, if there are *n*47 nucleotides in the lowest 47 sets, we compute F47 by *M*47/*D*47, where

(3) $$M47=\sum_{i=1}^{n47}m_{i}$$

and

(4) $$D47=\overset{n47}{\underset{i=1}{\sum }}d_{i}$$

The underpinning assumptions are that mixtures of biological origin are most likely to occur between two lineages, and, therefore, F2 is the most sensitive metric for identifying these. Since between 2 and 8 sets are mixed in such genuine coinfections, the lowest 47 (55 − 8) sets are not be mixed; thus, the F47 metric is more sensitive for identifying laboratory contamination involving more than two samples.

### Regression modeling.

Because of high leverage by a small number of observations, we used quantile regression to estimate the relationship between the median values of log-transformed noncallable base numbers and log-transformed F47, using the quantreg R package (R 3.3.1).

### Lineages to which the M. tuberculosis strains studied belong.

To describe the samples studied, we identified lineage using consensus basecalling in these 55 branches. If the signature SNV of a branch was called as uncertain, we called only to the level of the branch deeper (i.e., closer to the root) than the uncertain call. If more than one different lineage-defining variant was called or we could not call any lineage-defining positions, we reported the samples as “lineage not defined.” This process does not form part of the computation of F2 or F47 metrics.

### Ethics framework.

Only anonymized data were used in this work; therefore, approval from an ethics institution was not required.

### Data availability.

The data analyzed are available at https://ora.ox.ac.uk/objects/uuid:5e4ec1f8-e212-47db-8910-161a303a0757.

## RESULTS

### Samples studied.

A total of 4,156 samples were included since they were identified using MyKrobe (17) as belonging to the M. tuberculosis complex and had at least 0.5 × 10^6^ read pairs mapped to the H37Rv reference genome, a criterion reflecting successful DNA extraction and sequencing. These sequences were highly diverse, originating from six branches of lineage 1 (*n* = 320), five branches from lineage 2 (*n* = 278), five branches of lineage 3 (*n* = 1,010), and 30 branches of lineage 4 (*n* = 2,266). A total of 106 samples were from M. bovis or M. africanum, and 176 samples did not have their lineages defined (but were included in calculations of F2 and F47 metrics).

The laboratory processes operated under the following three different phases: in the first phase, development, laboratory processes were being actively refined; in the second phase, preproduction, laboratory processes were fixed and controlled by standard operating procedures, with version-controlled changes; and the third production state was similar to the second, except that the process had received ISO15189 accreditation.

### Variation in lineage-defining positions in H37Rv controls.

**FIG 2:**
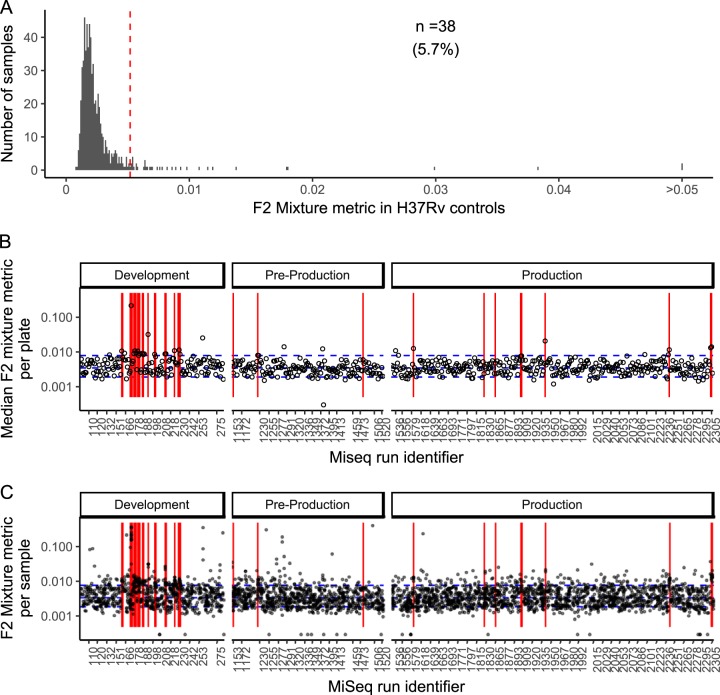
Mixtures in H37Rv controls. (A) Histogram showing the F2 metric, which reflects the mixture in the two most-mixed lineage-associated sets, in H37Rv control DNA. (B) Median F2 metric among clinical samples other than H37Rv; red lines indicate that the F2 mixture metric in H37Rv controls is raised (as shown in panel A). (C) F2 metric for each M. tuberculosis sequence from a clinical sample.

The F2 mixture metric reflects the estimated mixture in the two most-mixed lineage-defining sets; in the H37Rv controls, this follows a distribution skewed to the right (Fig. 2A). The MiSeq runs with H37Rv controls with F2 mixture metrics in the top 5% (Fig. 2A) are temporally clustered (red lines on Fig. 2B and C), with a number of examples in the development phase. Among clinical (noncontrol) samples, variation in the F2 metric is explained in part by the MiSeq run (Kruskal-Wallis test, *P* < 10^−16^), and a strong correlation exists between the F2 metric in H37Rv controls and that in clinical samples on the same plate (ρ = 0.61; 95% confidence interval [CI], 0.56 to 0.61; Spearman's rank correlation). That is, in plates with elevated F2 metrics in the H37Rv control, the clinical samples are more likely to have elevated F2, as is evident visually (see, e.g., Fig. 2B and C, around run 2,301).

### Different patterns of mixtures were observed during development.

**FIG 3:**
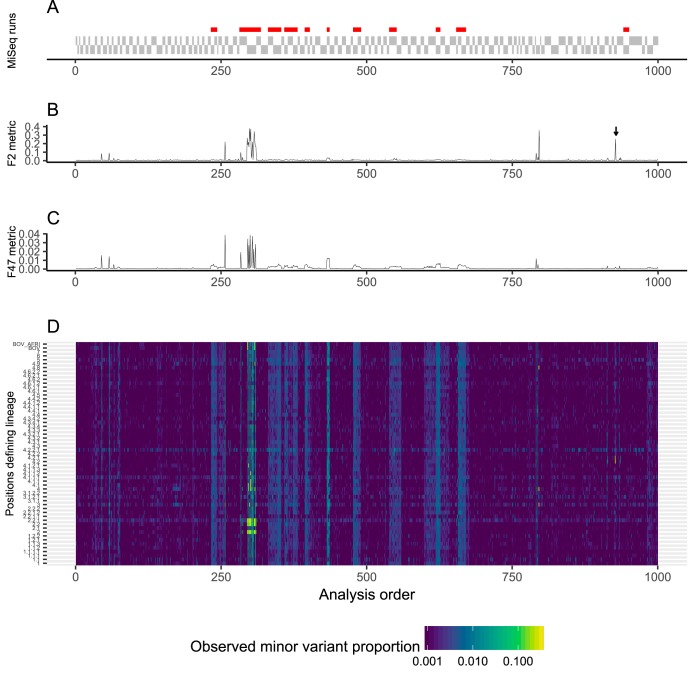
Mixture metrics in the development phase. (A) Samples are arranged first by the order of the MiSeq runs (depicted as solid gray blocks) and the order bioinformatics processing was completed. Only samples yielding M. tuberculosis are shown, which is why some blocks are longer than others. If the H37Rv control samples had increased F2 statistics, a red bar is shown above each sample in panel A. (B to D) We depicted the F2 (B) and F47 metrics (C), as well as the estimated mixture F in each of the 58 lineage-defining sets (D). The arrow illustrates a sample with elevated F2 but low F47 metrics.

We ordered specimens first by the order of the plates analyzed and the order in which the bioinformatics processing was completed, which is the order that an automated quality control monitoring system would encounter output. During the development phase (Fig. 3), blocks of samples derived from runs with elevated mixtures in the H37Rv control are seen (red bars in Fig. 3A), coincident with clear increases in both F2 and F47 metrics (Fig. 3B and C), reflecting elevations in mixed bases across most or all lineage-defining positions (Fig. 3D). These blocks of samples typically span multiple MiSeq runs (Fig. 3A and B). In addition to the blocks of samples with elevated F2 and F47 metrics, we also observed small numbers of single samples with elevated F2 but not F47 metrics (Fig. 3B, arrow). The pattern with single samples is expected in cases of interlineage mixtures of only two samples (see Fig. S1, Supplemental Methods). These patterns were also seen in the subsequent phases (see Fig. S2 to S5, yellow dots).

### Mixtures of multiple lineages are common.

Based on the pattern observed in the development phase, we categorized samples as having one of the following: (i) neither F2 nor F47 raised, (ii) raised F47, or (iii) raised F2 without raised F47. We defined a raised F2 and F47 as more than 10× and 5× the respective median metric during development in all control and clinical samples, cutoffs which correspond to 4.7% (F2) and 0.2% (F47) minor variant frequencies across the relevant lineage-defining sets, respectively. In the preproduction and production phases, F2 and F47 values below these thresholds (reflecting unmixed samples) were observed in 97.5% of the samples studied, raised F47 and F2 values (reflecting a mixture of multiple samples) were in observed 2.5% of the samples, and six samples (0.001%) had raised F2 but normal F47 values (Table 2).

**TABLE 2:** Detection of mixtures in clinical samples

Development stage (*n*)	No. (%) of mixtures with:		
	Neither F2 nor F47 raised	F2 raised, but F47 normal	F47 raised
Preproduction (919)	900 (98)	5 (0.003)	14 (1.1)
Production (1,794)	1,741 (97)	1 (0.001)	52 (2.8)

### Isolated F2 metric elevation is rare.

**FIG 4:**
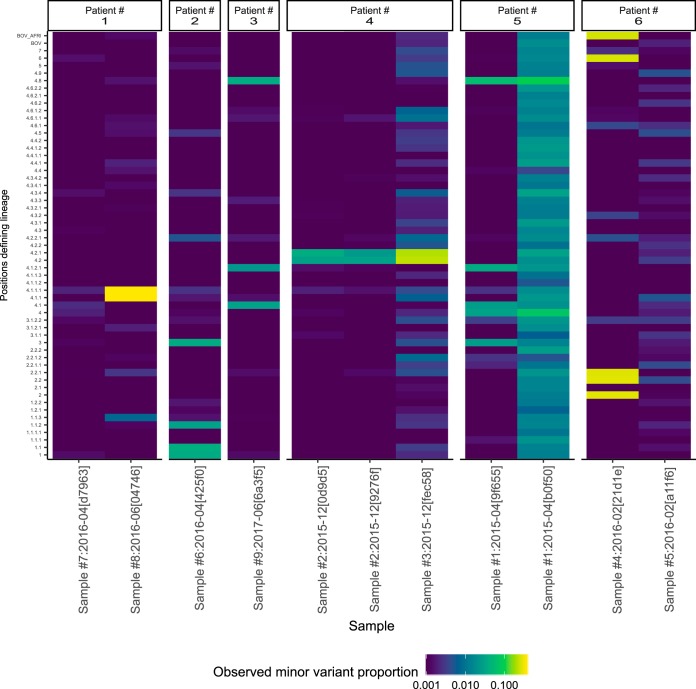
Consistency of isolated F2 elevation in individuals. Six individuals with elevated F2 but not F47 statistics were identified during the preproduction and production phases. The observed minor variant proportions for all deep branches analyzed are shown in a heatmap. For example, patient 4 had two samples taken in December 2015; sample 2 was analyzed twice (sequencing identification numbers [IDs] 0d9d5 and 9276f), and sample 3 was analyzed once. A similar pattern of minor variation is seen in all three samples.

Isolated elevation of F2 is expected if bacteria from two different lineages are mixed. In Fig. 4, we show the minor variant frequencies from all samples from the six individuals with raised F2 but normal F47 metrics. In one case, patient 3, two technical repeats of the same sample (sample 2) showed the same pattern, as did a separate sample taken contemporaneously. In other cases (patients 1, 5, and 6), the mixed pattern was only observed in one out of two positive samples taken on the same day, and in two cases (patients 2 and 3), only a single sample was positive. Thus, between 1 and 6 samples of the 4,156 samples studied may truly reflect mixed coinfections.

### Impact of interlineage variation on basecalling.

**FIG 5:**
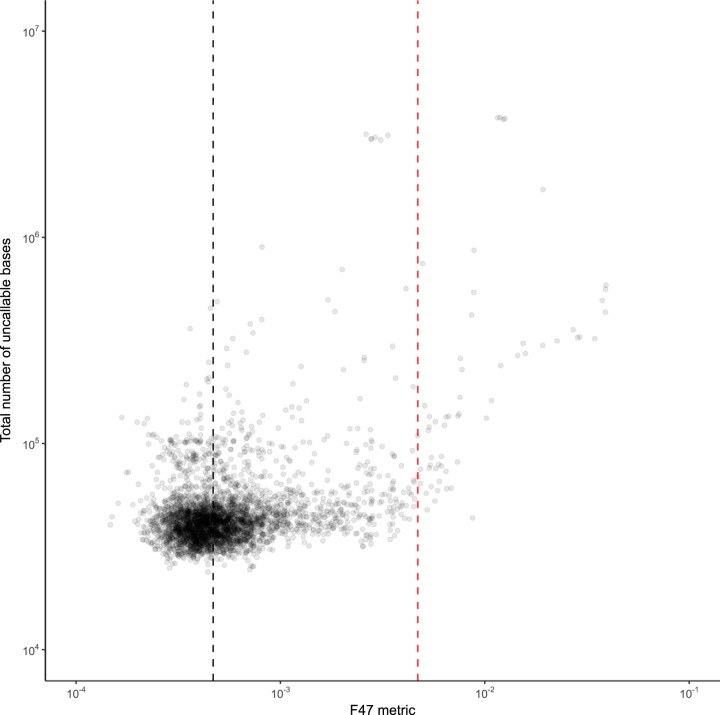
The relationship between the F47 metric and the number of uncallable bases is shown. The red line corresponds to the cutoff used to define F47 as being elevated.

One obvious question is whether very low-level cross-contamination impacts the consensus sequence which can discerned from the pileup. As cross-contamination increases, at some point minor variant frequencies in some parts of the genome will start to rise above the 10% cutoff specified by the basecalling algorithm. The numbers of uncalled bases will then rise; this relationship can be observed in Fig. 5, where the number of uncallable bases rises rapidly when F47 exceeds the cutoff value but only slowly below it. Below the cutoff value of 4.7% (red line in Fig. 5), which is 10× the median (black line in Fig. 5), the number of uncallable bases increased by 1.25-fold (95% CI, 1.22- to 1.28-fold) for every 10-fold increase in F47; above the cutoff, the corresponding increase was 9.24-fold (95% CI, 5.5- to 11.2-fold).

## DISCUSSION

In this work, we describe methods for monitoring the presence of mixtures of Mycobacterium tuberculosis of different lineages. The methods described are computationally efficient and will add little to the overall costs of sequencing and reporting. Our approach assumes that multiple lineages and sublineages of M. tuberculosis are being sequenced contemporaneously; this is the case in our setting and is also true globally (19, 20).

Using single-nucleotide variants, each of which uniquely defines a branch in the phylogenetic tree of M. tuberculosis, we can show two patterns of mixtures. The first, which occurred in about 2.5% of samples during the preproduction and production phases of our project, is indicative of multiple samples being mixed together, since mixtures are seen in most or all of the lineage-defining branches. This occurred in batches, was characterized by cross-contamination at levels of less than 1%, and can be monitored by a metric we term F47. This pattern likely reflects process failures. The strength of the F47 metric is that the depth analyzed is very high, as about 5,000 nucleotides typically contribute across the lineage-defining sets included in it. If there is a sequencing depth of 50 to 100 reads at each of these, the effective sequencing depth analyzed is of the order of 25,000 to 50,000 reads, making the detection of minor variations at sub-1% levels readily feasible with high statistical confidence. Possible points of cross-contamination include DNA extraction, which occurs in batches, and library preparation, which also occurs in batches. Low-level cross-contamination during centrifugation of a batch of samples in a microcentrifuge is one possibility. Whether such cross-contamination would be expected to affect the H37Rv internal control depends on whether this control is processed end to end with each batch or whether H37Rv DNA is preprepared and added at the library construction stage.

Such low-level cross-contamination, as observed during our production process and illustrated in Fig. S2 to S4, is likely to have minimal influence on inference drawn from the sequence, unless highly sensitive assays for heteroresistance are required. However, given the rarity of detection of intersample mixtures during production use of M. tuberculosis sequencing, repeating extraction and sequencing of samples in batches with elevated F47 metrics could be considered. A sensitive metric, such as F47, will also allow the early detection of emerging problems and allow a review of the process as part of continuous quality improvement.

A second class of mixture, which was rarely detected in this setting, is compatible with coinfection with two organisms of differing lineages within the patient. This kind of mixture is clinically relevant (7, 11) and may be underdetected using the laboratory process we describe here, since culture-based amplification can reduce diversity in the sample inoculated (10). Its frequency may rise if direct-from-sample sequencing is employed or if samples from areas with high endemicity are studied, but here, we identified only one probable case of such mixtures and five other possible cases. Confirmatory approaches are available; microbiological techniques conducted separately on multiple picks from the same samples have been used as validation (3). A limitation of this study is that we could not undertake such work, as only multiply subcultured stored isolates exist for historical samples. Techniques for reconstructing the contributing sequences also have been described in detail (21–23), and we did not study them here. Another limitation is that we were not able to consider six of the lineage-defining sets in the study by Coll et al., because they covered <20 nucleotide positions; therefore, we considered that they did not contain sufficient information to be used in F2 or F47 metrics. A consequence is that our method did not identify mixtures of samples if they only involved mixtures in these excluded branches. Practically, such observations should be assessed on a case-by-case basis, having reviewed results from other samples from the same patient, treatment history, and basecalls at resistance loci for possible heteroresistance. More intensive follow-up could be considered (3), but there are few data to define optimal management.

The clinical use of bacterial genome sequencing is rising (16, 17, 24), and given the importance of M. tuberculosis and the complexity of treatment, M. tuberculosis has been one of the first organisms tested in such a way (15). The processes followed involve multiple steps at which the opportunity for cross-contamination exists. The availability of tools monitoring the critical aspects of the laboratory process is required for accreditation under ISO15189, and the F2 and F47 metrics described here will contribute to addressing this gap.

## Supplementary Material

Supplemental file 1

## References

[1] Comas I, Coscolla M, Luo T, Borrell S, Holt KE, Kato-Maeda M, Parkhill J, Malla B, Berg S, Thwaites G, Yeboah-Manu D, Bothamley G, Mei J, Wei L, Bentley S, Harris SR, Niemann S, Diel R, Aseffa A, Gao Q, Young D, Gagneux S 2013 Out-of-Africa migration and Neolithic coexpansion of Mycobacterium tuberculosis with modern humans. Nat Genet 45:1176–1182. doi:10.1038/ng.2744. 23995134 PMC3800747

[2] Coll F, McNerney R, Guerra-Assuncao JA, Glynn JR, Perdigao J, Viveiros M, Portugal I, Pain A, Martin N, Clark TG 2014 A robust SNP barcode for typing Mycobacterium tuberculosis complex strains. Nat Commun 5:4812. doi:10.1038/ncomms5812. 25176035 PMC4166679

[3] Cohen T, van Helden PD, Wilson D, Colijn C, McLaughlin MM, Abubakar I, Warren RM 2012 Mixed-strain Mycobacterium tuberculosis infections and the implications for tuberculosis treatment and control. Clin Microbiol Rev 25:708–719. doi:10.1128/CMR.00021-12. 23034327 PMC3485752

[4] Lieberman TD, Wilson D, Misra R, Xiong LL, Moodley P, Cohen T, Kishony R 2016 Genomic diversity in autopsy samples reveals within-host dissemination of HIV-associated Mycobacterium tuberculosis. Nat Med 22:1470–1474. doi:10.1038/nm.4205. 27798613 PMC5508070

[5] Bryant JM, Harris SR, Parkhill J, Dawson R, Diacon AH, van Helden P, Pym A, Mahayiddin AA, Chuchottaworn C, Sanne IM, Louw C, Boeree MJ, Hoelscher M, McHugh TD, Bateson AL, Hunt RD, Mwaigwisya S, Wright L, Gillespie SH, Bentley SD 2013 Whole-genome sequencing to establish relapse or re-infection with Mycobacterium tuberculosis: a retrospective observational study. Lancet Respir Med 1:786–792. doi:10.1016/S2213-2600(13)70231-5. 24461758 PMC3861685

[6] Wang X, Liu H, Wei J, Wu X, Yu Q, Zhao X, Lyu J, Lou Y, Wan K 2015 An investigation on the population structure of mixed infections of Mycobacterium tuberculosis in Inner Mongolia, China. Tuberculosis (Edinb) 95:695–700. doi:10.1016/j.tube.2015.08.006. 26542224

[7] Cohen T, Chindelevitch L, Misra R, Kempner ME, Galea J, Moodley P, Wilson D 2016 Within-host heterogeneity of Mycobacterium tuberculosis infection is associated with poor early treatment response: a prospective cohort study. J Infect Dis 213:1796–1799. doi:10.1093/infdis/jiw014. 26768249 PMC4857469

[8] Kay GL, Sergeant MJ, Zhou Z, Chan JZ, Millard A, Quick J, Szikossy I, Pap I, Spigelman M, Loman NJ, Achtman M, Donoghue HD, Pallen MJ 2015 Eighteenth-century genomes show that mixed infections were common at time of peak tuberculosis in Europe. Nat Commun 6:6717. doi:10.1038/ncomms7717. 25848958 PMC4396363

[9] Chan JZ, Sergeant MJ, Lee OY, Minnikin DE, Besra GS, Pap I, Spigelman M, Donoghue HD, Pallen MJ 2013 Metagenomic analysis of tuberculosis in a mummy. N Engl J Med 369:289–290. doi:10.1056/NEJMc1302295. 23863071

[10] Plazzotta G, Cohen T, Colijn C 2015 Magnitude and sources of bias in the detection of mixed strain M. tuberculosis infection. J Theor Biol 368:67–73. doi:10.1016/j.jtbi.2014.12.009. 25553967 PMC7011203

[11] Zetola NM, Modongo C, Moonan PK, Ncube R, Matlhagela K, Sepako E, Collman RG, Bisson GP 2014 Clinical outcomes among persons with pulmonary tuberculosis caused by Mycobacterium tuberculosis isolates with phenotypic heterogeneity in results of drug-susceptibility tests. J Infect Dis 209:1754–1763. doi:10.1093/infdis/jiu040. 24443546 PMC4017367

[12] Walker TM, Lalor MK, Broda A, Ortega LS, Morgan M, Parker L, Churchill S, Bennett K, Golubchik T, Giess AP, Del Ojo Elias C, Jeffery KJ, Bowler I, Laurenson IF, Barrett A, Drobniewski F, McCarthy ND, Anderson LF, Abubakar I, Thomas HL, Monk P, Smith EG, Walker AS, Crook DW, Peto TEA, Conlon CP 2014 Assessment of Mycobacterium tuberculosis transmission in Oxfordshire, UK, 2007-12, with whole pathogen genome sequences: an observational study. Lancet Respir Med 2:285–292. doi:10.1016/S2213-2600(14)70027-X. 24717625 PMC4571080

[13] Walker TM, Ip CL, Harrell RH, Evans JT, Kapatai G, Dedicoat MJ, Eyre DW, Wilson DJ, Hawkey PM, Crook DW, Parkhill J, Harris D, Walker AS, Bowden R, Monk P, Smith EG, Peto TE 2013 Whole-genome sequencing to delineate Mycobacterium tuberculosis outbreaks: a retrospective observational study. Lancet Infect Dis 13:137–146. doi:10.1016/S1473-3099(12)70277-3. 23158499 PMC3556524

[14] Guerra-Assuncao JA, Houben RM, Crampin AC, Mzembe T, Mallard K, Coll F, Khan P, Banda L, Chiwaya A, Pereira RP, McNerney R, Harris D, Parkhill J, Clark TG, Glynn JR 2015 Recurrence due to relapse or reinfection with Mycobacterium tuberculosis: a whole-genome sequencing approach in a large, population-based cohort with a high HIV infection prevalence and active follow-up. J Infect Dis 211:1154–1163. doi:10.1093/infdis/jiu574. 25336729 PMC4354982

[15] Quan TP, Bawa Z, Foster D, Walker T, Del Ojo Elias C, Rathod P, Iqbal Z, Bradley P, Mowbray J, Walker AS, Crook DW, Wyllie DH, Peto TEA, Smith EG 2017 Evaluation of whole-genome sequencing for Mycobacterial species identification and drug susceptibility testing in a clinical setting: a large-scale prospective assessment of performance against line-probe assays and phenotyping. J Clin Microbiol 56:e01480-17. doi:10.1128/jcm.01480-17. PMC5786738 29167290

[16] Pankhurst LJ, Del Ojo Elias C, Votintseva AA, Walker TM, Cole K, Davies J, Fermont JM, Gascoyne-Binzi DM, Kohl TA, Kong C, Lemaitre N, Niemann S, Paul J, Rogers TR, Roycroft E, Smith EG, Supply P, Tang P, Wilcox MH, Wordsworth S, Wyllie D, Xu L, Crook DW 2016 Rapid, comprehensive, and affordable mycobacterial diagnosis with whole-genome sequencing: a prospective study. Lancet Respir Med 4:49–58. doi:10.1016/S2213-2600(15)00466-X. 26669893 PMC4698465

[17] Bradley P, Gordon NC, Walker TM, Dunn L, Heys S, Huang B, Earle S, Pankhurst LJ, Anson L, de Cesare M, Piazza P, Votintseva AA, Golubchik T, Wilson DJ, Wyllie DH, Diel R, Niemann S, Feuerriegel S, Kohl TA, Ismail N, Omar SV, Smith EG, Buck D, McVean G, Walker AS, Peto TE, Crook DW, Iqbal Z 2015 Rapid antibiotic-resistance predictions from genome sequence data for Staphylococcus aureus and Mycobacterium tuberculosis. Nat Commun 6:10063. doi:10.1038/ncomms10063. 26686880 PMC4703848

[18] Lunter G, Goodson M 2011 Stampy: a statistical algorithm for sensitive and fast mapping of Illumina sequence reads. Genome Res 21:936–939. doi:10.1101/gr.111120.110. 20980556 PMC3106326

[19] Stucki D, Brites D, Jeljeli L, Coscolla M, Liu Q, Trauner A, Fenner L, Rutaihwa L, Borrell S, Luo T, Gao Q, Kato-Maeda M, Ballif M, Egger M, Macedo R, Mardassi H, Moreno M, Tudo Vilanova G, Fyfe J, Globan M, Thomas J, Jamieson F, Guthrie JL, Asante-Poku A, Yeboah-Manu D, Wampande E, Ssengooba W, Joloba M, Henry Boom W, Basu I, Bower J, Saraiva M, Vaconcellos SEG, Suffys P, Koch A, Wilkinson R, Gail-Bekker L, Malla B, Ley SD, Beck HP, de Jong BC, Toit K, Sanchez-Padilla E, Bonnet M, Gil-Brusola A, Frank M, Penlap Beng VN, Eisenach K, Alani I, Wangui Ndung'u P, 2016 Mycobacterium tuberculosis lineage 4 comprises globally distributed and geographically restricted sublineages. Nat Genet 48:1535–1543. doi:10.1038/ng.3704. 27798628 PMC5238942

[20] Manson AL, Cohen KA, Abeel T, Desjardins CA, Armstrong DT, Barry CE III, Brand J, Chapman SB, Cho SN, Gabrielian A, Gomez J, Jodals AM, Joloba M, Jureen P, Lee JS, Malinga L, Maiga M, Nordenberg D, Noroc E, Romancenco E, Salazar A, Ssengooba W, Velayati AA, Winglee K, Zalutskaya A, Via LE, Cassell GH, Dorman SE, Ellner J, Farnia P, Galagan JE, Rosenthal A, Crudu V, Homorodean D, Hsueh PR, Narayanan S, Pym AS, Skrahina A, Swaminathan S, Van der Walt M, Alland D, Bishai WR, Cohen T, Hoffner S, Birren BW, Earl AM 2017 Genomic analysis of globally diverse Mycobacterium tuberculosis strains provides insights into the emergence and spread of multidrug resistance. Nat Genet 49:395–402. doi:10.1038/ng.3767. 28092681 PMC5402762

[21] Eyre DW, Cule ML, Griffiths D, Crook DW, Peto TE, Walker AS, Wilson DJ 2013 Detection of mixed infection from bacterial whole genome sequence data allows assessment of its role in Clostridium difficile transmission. PLoS Comput Biol 9:e1003059. doi:10.1371/journal.pcbi.1003059. 23658511 PMC3642043

[22] Pulido-Tamayo S, Sanchez-Rodriguez A, Swings T, Van den Bergh B, Dubey A, Steenackers H, Michiels J, Fostier J, Marchal K 2015 Frequency-based haplotype reconstruction from deep sequencing data of bacterial populations. Nucleic Acids Res 43:e105. doi:10.1093/nar/gkv478. 25990729 PMC4652744

[23] Gan M, Liu Q, Yang C, Gao Q, Luo T 2016 Deep whole-genome sequencing to detect mixed infection of Mycobacterium tuberculosis. PLoS One 11:e0159029. doi:10.1371/journal.pone.0159029. 27391214 PMC4938208

[24] De Silva D, Peters J, Cole K, Cole MJ, Cresswell F, Dean G, Dave J, Thomas DR, Foster K, Waldram A, Wilson DJ, Didelot X, Grad YH, Crook DW, Peto TE, Walker AS, Paul J, Eyre DW 2016 Whole-genome sequencing to determine transmission of Neisseria gonorrhoeae: an observational study. Lancet Infect Dis 16:1295–1303. doi:10.1016/S1473-3099(16)30157-8. 27427203 PMC5086424

