# Supplementary material for: Identifying Mixed Mycobacterium tuberculosis Infection and Laboratory Cross-Contamination during Mycobacterial Sequencing Programs
Source: J Clin Microbiol. 2018 Oct 25;56(11):e00923-18. doi: 10.1128/JCM.00923-18 (PMC6204665; doi:10.1128/JCM.00923-18)
Supplement: Supplemental file 1 [file zjm999096152s1.pdf]

Supplementary data for publication

Supplementary Figures

Supplementary Figure 1

Illustrates the computation of variation between samples of two different lineages, Lineage 1 (black text) and Lineage 2 (blue text) (A). When a mixture of these samples is present, and mapped to a reference sequence, a major base and minor base(s) are present in the pileup (B). Variation may be due to either sequencing error (underlined) or to lineage associated variation; the non-major variant frequency included both classes of variation. Lineage defining sites, as defined by Coll *et al* (1), mark branches of the phylogenetic tree. If a lineage 4.1.1.1 *M. tuberculosis* is mixed with a lineage 3.1.2.1 *M. tuberculosis*, eight sets of lineage defining sites will be mixed (red boxes).

Supplementary Figures 2-5

These illustrate mixture patterns observed during the Production stage. The layout is similar to Figure 3; samples arranged first by the order of the MiSeq runs (depicted as solid gray blocks, in A), and the order bioinformatics processing completed. Only samples yielding *M. tuberculosis* samples are shown, which is why some blocks in A are longer than others. If the H37Rv control samples had increase F2 statistics, a red bar is shown above each sample in A. We depicted the F2 metric (B) and F47 metrics (C), as well as the estimated mixture F in each of the 58 lineage defining sets (D).

References

1. Coll F, McNerney R, Guerra-Assuncao JA, Glynn JR, Perdigao J, Viveiros M, Portugal I, Pain A, Martin N, Clark TG. 2014. A robust SNP barcode for typing Mycobacterium tuberculosis complex strains. Nat Commun 5:4812.

Supp. Fig. 1

A

Sequence of

ACATACGTACGTACGTACGT — lineage 1

ACGTACGT**T**C**A**TACGTACGT — lineage 2

Reads from

ACATACGTACGT
GTACGTACGTACGT
CGTACGTACTTACGT
GTACGTACGTACGT
ACGTACGT**T**C**A**TACGTACGT
AAGTACGT**T**C**A**TACGTCCG
CGGACGT**T**C**A**TACGTACGT

lineage 1
lineage 2

T (bold) lineage defining variant

T (underlined) Variation due to error

B

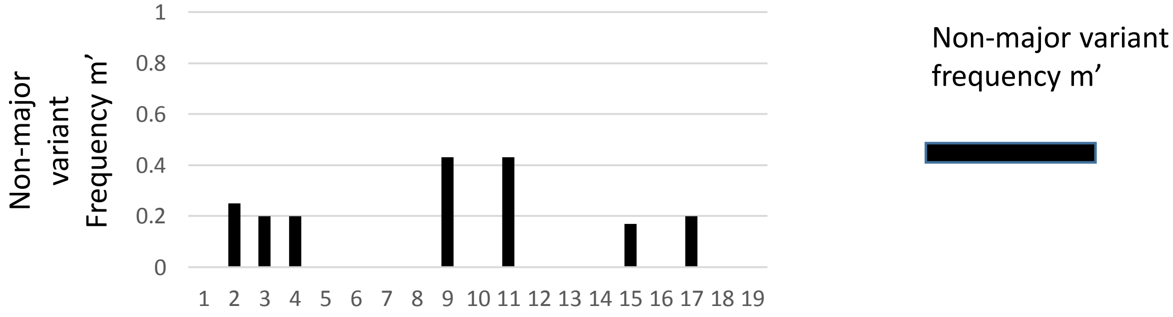

C

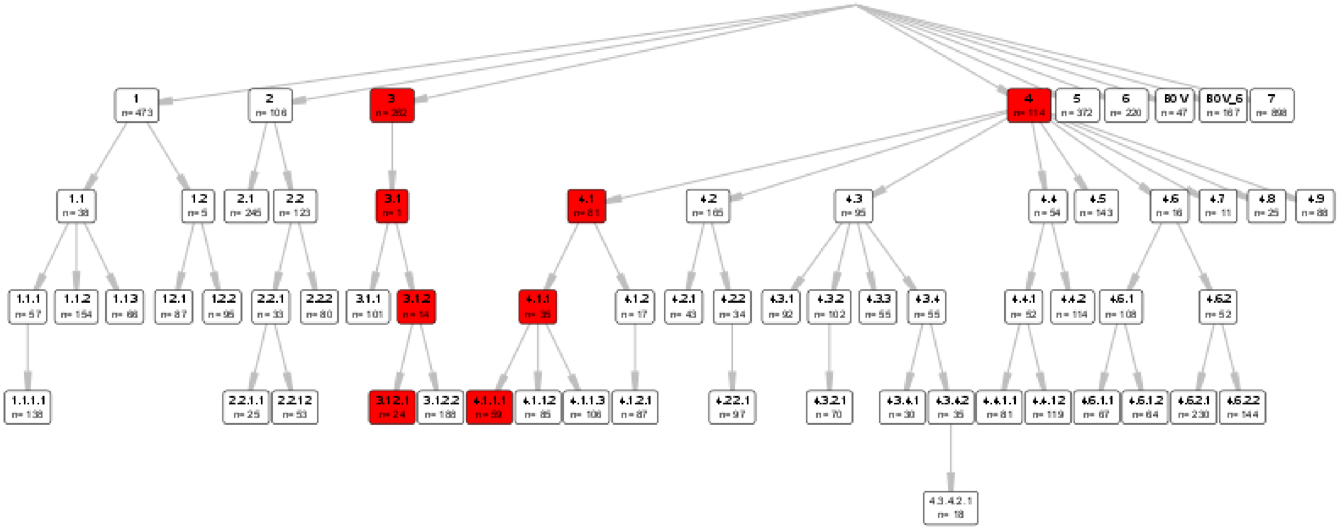

Supp. Fig. 2

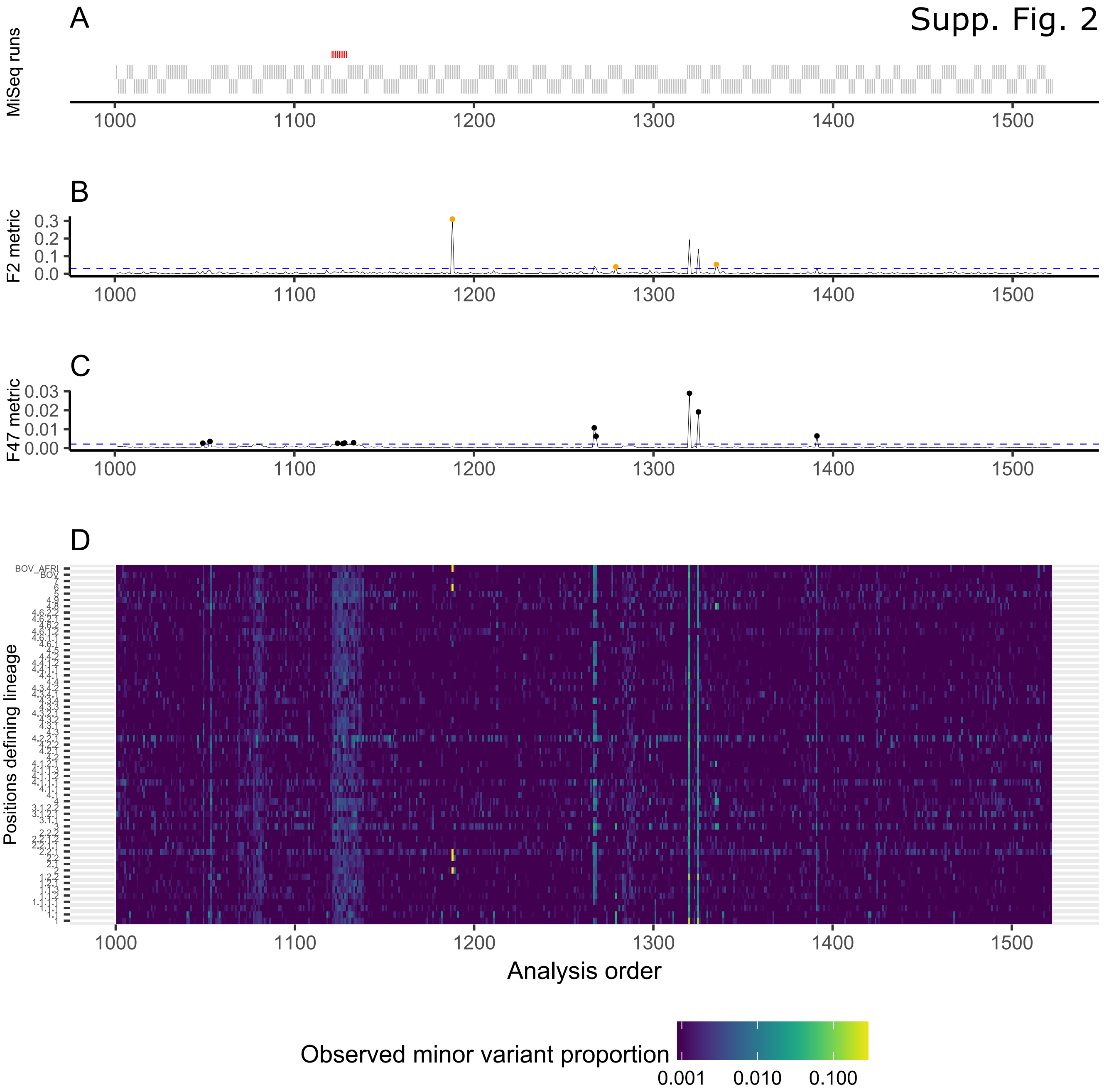
A
MiSeq runs
B
F2 metric
C
F47 metric
D
Positions defining lineage
BOV_AFRI
BOV
Analysis order
Observed minor variant proportion
0.001
0.010
0.100
1000
1100
1200
1300
1400
1500

Supp. Fig. 3

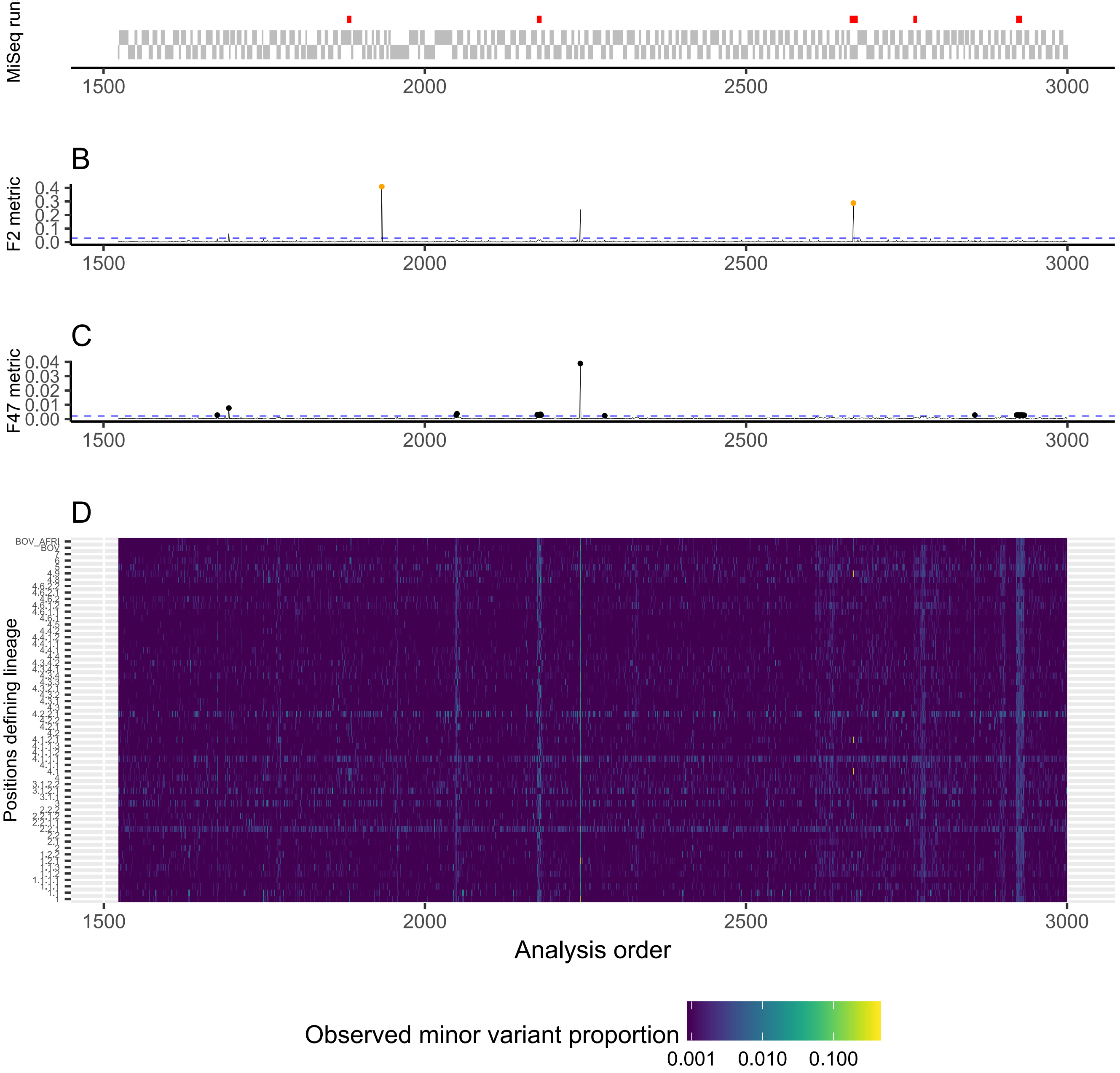

A
MiSeq runs
1500
2000
2500
3000
B
F2 metric
0.4
0.3
0.2
0.1
0.0
C
F47 metric
0.04
0.03
0.02
0.01
0.00
D
Positions defining lineage
BOV_AFRI
BOV
Analysis order
Observed minor variant proportion
0.001
0.010
0.100

Supp. Fig. 4

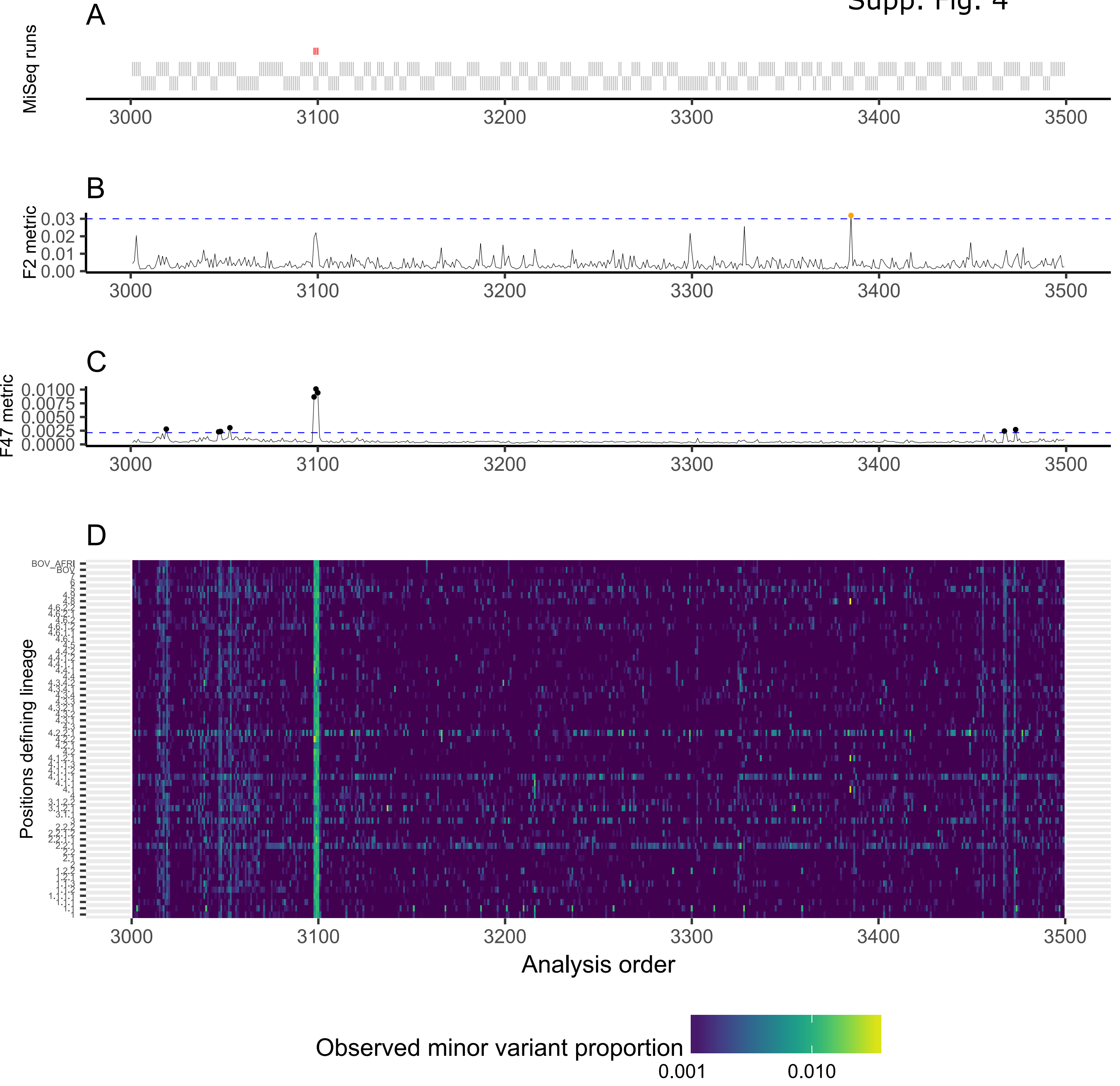

A
MiSeq runs
B
F2 metric
C
F47 metric
D
Positions defining lineage
Analysis order
Observed minor variant proportion
0.001
0.010

Supp. Fig. 5

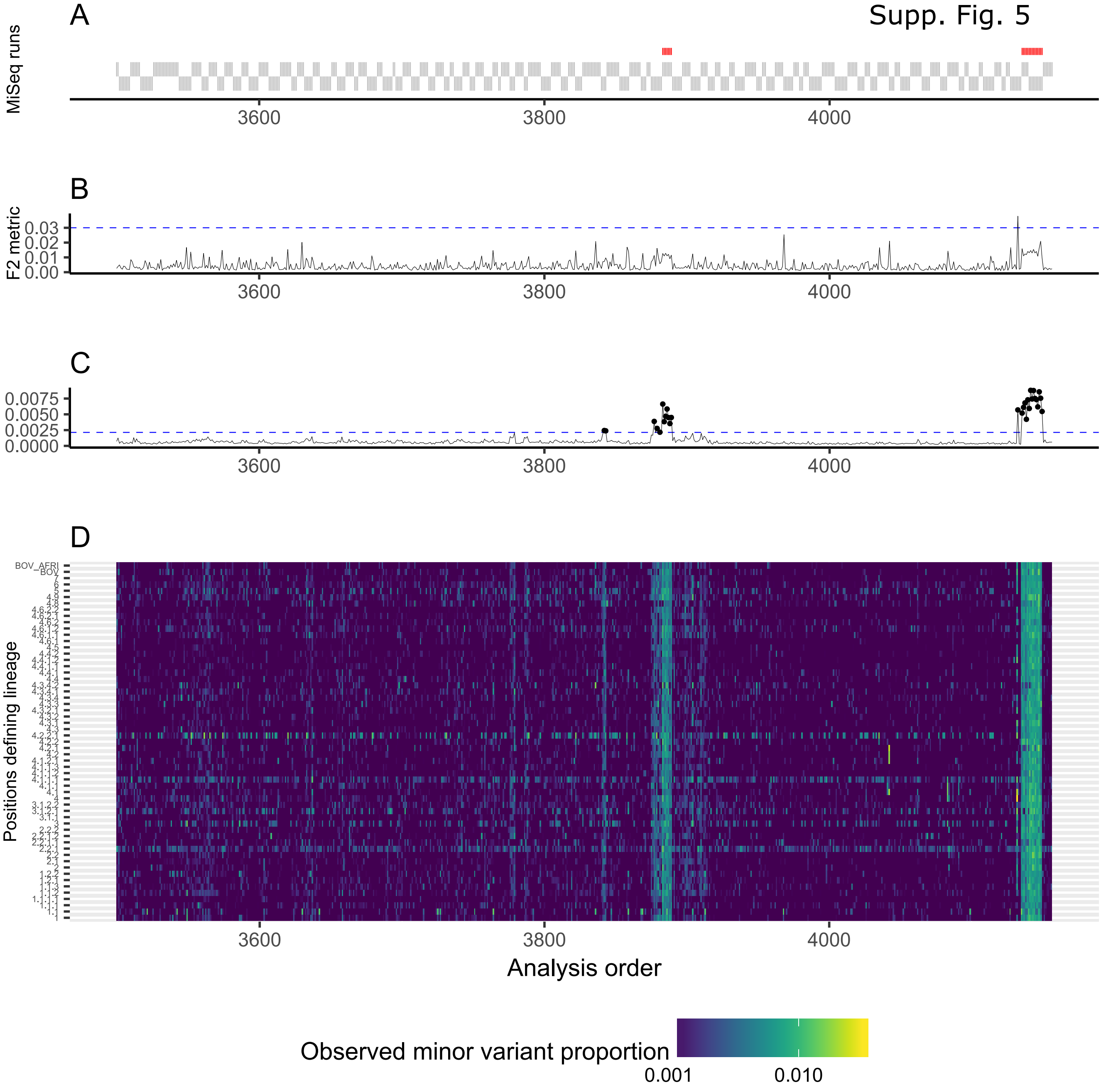
A
MiSeq runs
3600
3800
4000
B
F2 metric
0.03
0.02
0.01
0.00
C
0.0075
0.0050
0.0025
0.0000
D
Positions defining lineage
BOV_AFRI
BOV
Analysis order
Observed minor variant proportion
0.001
0.010
